# Medical management of overactive bladder

**DOI:** 10.4103/0970-1591.65403

**Published:** 2010

**Authors:** Sarvpreet S. Ubee, Ramaswamy Manikandan, Gurpreet Singh

**Affiliations:** Department of Urology, Royal Liverpool University Hospital, Liverpool, United Kingdom; 1 Wrightington, Wigan and Leigh NHS Foundation Trust, Wigan, UK; 2 Southport District and General Hospital, Southport, UK

**Keywords:** Antimuscarinics, bladder training, overactive bladder

## Abstract

Overactive bladder (OAB), as defined by the International Continence Society, is characterized by a symptom complex including urinary urgency with or without urge incontinence, usually associated with frequency and nocturia. OAB syndrome has an incidence reported from six European countries ranging between 12-17%, while in the United States; a study conducted by the National Overactive Bladder Evaluation program found the incidence at 17%. In Asia, the prevalence of OAB is reported at 53.1%. In about 75%, OAB symptoms are due to idiopathic detrusor activity; neurological disease, bladder outflow obstruction (BOO) intrinsic bladder pathology and other chronic pelvic floor disorders are implicated in the others. OAB can be diagnosed easily and managed effectively with both non-pharmacological and pharmacological therapies. The first-line treatments are lifestyle interventions, bladder training, pelvic floor muscle exercises and anticholinergic drugs. Antimuscarinics are the drug class of choice for OAB symptoms; with proven efficacy, and adverse event profiles that differ somewhat.

## INTRODUCTION

Overactive bladder (OAB), as defined by the International Continence Society (ICS), is characterized by a symptom complex including urinary urgency with or without urge incontinence, usually associated with frequency and nocturia.[1] This is distinct from the urodynamic diagnosis of detrusor overactivity (DO); ICS defines this as an involuntary rise in detrusor pressure during filling of the bladder in a laboratory situation in a conscious co-operative patient.[1]

OAB syndrome has an incidence reported from six European countries ranging between 12-17%, while in the United States; a study conducted by the National Overactive Bladder Evaluation (NOBLE) program found the incidence at 17%.[2–4] In Asia, the prevalence of OAB is reported at 53.1%.[5] The prevalence of OAB increases with age.[2] Storage Lower Urinary Tract Symptoms (LUTS) comprise OAB and are extremely bothersome, especially with urge incontinence (UI) coexistent in 30% of patients. The etiology is unknown, and it is suggested that OAB symptoms could be either neurogenic or myogenic. The neurogenic theory suggests that damage to central inhibitory pathways or sensitization of afferent pathways in the bladder unmasks primitive voiding reflexes and trigger detrusor overactivity.[6] Myogenic theory proposes a partial denervation of the detrusor with an increased excitability and involuntary pressure rise.[7] A third, the autonomous bladder hypothesis has recently been proposed; this suggests that overactivity may be due to inappropriate activation or modulation of phasic activity.[8]

In about 75%, OAB symptoms are due to idiopathic detrusor activity (IDO); neurological disease, bladder outflow obstruction (BOO) intrinsic bladder pathology and other chronic pelvic floor disorders are implicated in the others.

## ASSESSMENT AND OUTCOME STRATEGY

OAB symptoms are subjective; with an unpredictable relationship to their bothersome index. Based on the history alone, clinicians tend to underestimate the impact of symptoms and patient completed assessment methods are essential.[9] Both subjective and objective assessment methods measure different aspects of a patient’s clinical profile and should be interpreted as assessing a different outcome.[10] The overall patient experience can be better understood by identifying objective treatment endpoints; including changes in the quality of life, symptom bother, and patient satisfaction.

A wide range of tools have been developed for use in the clinical settings. Validated disease-specific quality of life questionnaires like the International Consultation on Incontinence (ICIQ) assess the level of bother; frequency-volume charts assess voiding and drinking patterns. A common method of objective assessment is the bladder diary, which records the voiding times, volumes, incontinence episodes, pad usage, fluid intake, degree of urgency and degree of incontinence. Synchronous pressure flow studies are mandatory for objective assessment. Reflex volumes, amplitude of involuntary detrusor contractions, bladder compliance and maximum cystometric capacity along with voiding detrusor pressures and maximum urinary flow rate are important urodynamic parameters.

## INITIAL MANAGEMENT – GUIDELINES

ICS has guidelines subdividing management into initial (first line) and specialized (second line) therapy. Non-surgical treatment is the mainstay and should include lifestyle, including dietary modifications, bladder retraining and pelvic floor training with or without biofeedback; these are combined with pharmacotherapy with antimuscarinics as appropriate.

## LIFESTYLE INTERVENTIONS DIETARY AND FLUID MODIFICATION

Epidemiological evidence suggests that reducing bladder irritants such as acidic foods, alcohol, and caffeine may improve urinary incontinence.[11] A study of 4000 men examining the role of food and alcohol in the development of OAB symptoms found that alcohol had a protective effect; and of the foods, only potatoes appeared to have a detrimental effect on symptoms.[12] Another study of 6,424 women found that smoking, obesity and consumption of carbonated beverages were associated with OAB; alcohol did not make a difference.[13]

Research on the effect of caffeine has been conflicting; one of the studies showed a decrease in frequency with caffeine withdrawal and another did not show any statistically significant association between caffeine intake and urinary incontinence.[14,15]

Many patients with OAB attempt to restrict their fluid intake, in a belief that it will reduce their incontinence episodes. However, dehydration and concentrated urine can act as a bladder irritant; studies have demonstrated a strong association between fluid intake and voided volume.[16,17] The association of fluid intake with incontinence episodes was weak, but decreasing evening fluid intake reduced nocturnal incontinence.

## BLADDER TRAINING

The goal of bladder training is to break the cycle of urgency and frequency using consistent, incremental voiding schedules. To accomplish this, the brain must be trained to ignore the signals from the detrusor muscle. It is hypothesized that urinary urgency develops first and this leads to urinary frequency. As this cycle progresses, the patient develops a decreased bladder capacity, which will then lead to detrusor overactivity.[18,19]

The patient fills in a bladder diary; recording the times of micturition, voided volumes, urgency and incontinence episodes, pad usage, and fluid intake. The duration of the diary can range from one to seven days, with a four-day diary as reliable as a seven-day diary.[20] A minimum of three days need recording, to give the frequency volume chart validity.[21] Using this data, the voiding and fluid intake habits are modified to increase the bladder capacity. Fantl *et al*. in a randomized clinical trial showed a 57% reduction in incontinence episodes in older women; there was, however, no objective improvement on urodynamic parameters post bladder training.[22] Quoted results vary from 26 to 90% and bladder training remains a valuable tool for treating urge incontinence as it weakens the urge-void response.[23,24]

### Pelvic floor muscle exercises and biofeedback

Pelvic floor muscle exercises (PFME), originally described by Kegel in 1948 for stress incontinence, may also reduce urge incontinence episodes.[25] In a study by Burgio *et al*. 197 women with urge incontinence were randomized in to three groups. Patients on PFME with biofeedback had an 80.7% reduction in incontinent episodes compared to a 68.5% and a 39.4% reduction in patients on medical therapy and placebo.[26] Three studies randomized patients with mixed incontinence to PFME, PFME with bladder training and control groups. The two groups utilizing PFME had improvement ranging from 54 to 74% in comparison to the control group.[27–29] Though most of the trials are on patients with mixed and stress incontinence, a Cochrane Meta-analysis concluded that PFME is valid first line therapy for urinary incontinence.[30]

Biofeedback ensures that with PFME, the right muscle groups are being exercised; and can vary from verbal feedback to vaginal or anal electromyography. A randomized study showed a 69.4, 63.1; and 58.5% reduction in incontinence with verbal feedback, anal myometry feedback and in controls, with the difference between the three groups not reaching statistical significance.[31]

### Pharmacotherapy

Antimuscranics are established first line treatment options for OAB symptoms. In randomized controlled trials they are more effective than placebo as shown in a recent meta-analysis.[32] The level of recommendation is grade A in women and grade B in men with OAB.[33] Despite good efficacy antimuscarinics have significant side effects and compliance with treatment is at best average.

### Muscarinic receptors

Acetylcholine activates muscarinic receptors on detrusor myocytes and is the main contractile transmitter in the bladder. Five subtypes, of Muscarinic receptors (M1–M5), encoded by 5 distinct genes are identified. In the human detrusor smooth muscle M2 and M3 subtypes predominate, with M3 as the most important for detrusor contraction.[34] Studies have shown that the M3 receptors work by mediating carbacol-induced contractions and it’s suggested that transmembrane flux of Ca++ through nifedepine – sensitive calcium channels as well as activation of the Rho-kinase pathway and inhibition of potassium channel, may be implicated. It was demonstrated that the L-type calcium channel blocker nifedipine almost completely inhibited carbachol-induced detrusor contraction.[9,35]

A functional role for the M2 receptor has not been definitely identified and the hypothesis is that; it opposes sympathetically mediated smooth muscle relaxation.[36]

### Antimuscarinics

It is suggested to reduce spontaneous activity in the resting detrusor muscle, thus decreasing the frequency and intensity of the detrusor contractions.[10,37] Yokoyama *et al*., in a study on rats showed that tolteradine exerted an inhibitory effect on the C-fiber afferent bladder nerves, thereby improving the bladder capacity.[38] *In vitro* and clinical studies have shown that the effects are due to its action on the storage phase.[39,40]

Propantheline, methantheline, emepronium, diclomine, terodiline and oxybutynin are antimuscarinics historically used to treat OAB; of these oxybutynin remains in current use, the others discontinued due to lack of efficacy and/or poor tolerability.[10] Newer agents like propiverine, tolterodine, trospium, solifenacin and darifenacin along with oxybutynin have proven efficacy in patients with OAB.[40] These antimuscarinics are available in various formulations like immediate release, extended release and transdermal preparations. The extended-release form allows steady plasma concentrations for up to 24 hours and minimizes the peak and trough concentrations seen with conventional multiple-daily dosage form.[41] Transdermal delivery of oxybutynin decreases the associated side effects as it avoids the hepatic and gastrointestinal metabolism, producing less N-desethyloxybutynin, a metabolite, deemed responsible for side-effects such as dry mouth.[42]

These antimuscarinics have variable affinity to the muscarinic receptors sub-types. Oxybutynin and solifenacin are moderately selective for M3 relative to M2 receptors; and darifenacin is selective for M3 relative to M2, and to a lesser extent to the M1 receptors. The pharmacokinetics of the antimuscarinics varies considerably [Table 1].

**Table 1:** Pharmacokinetics of antimuscarinics

	Oxybutynin IR/ER	Transdermal oxybutynin	Tolterodine	Solifenacin	Trospium	Darifenacin	Properavine
Molecular weight	393.19	357	475.6	480.55	427.97	507.5	403.95
Lipophilicity	Lipophilic	Lipophilic	Slightly lipophilic	Lipophilic	Hydrophilic	Highly lipophilic	NA
Metabolizing enzymes	CYP3A4	CYP3A4	CYP2D6, CYP3A4	CYP3A4	NonCYP450 hydrolysis	CYP2D6, CYP3A4	CYP2D6, CYP3A4
Active metabolites	des-ethyl-oxybutynin	des-ethyl-oxybutynin	5-hydroxy-methlytolterodine	None	None	None	propverine N-oxide
Half life	2 /13 hrs	7-8 hrs	2/8 hrs	45-68 hrs	20 hrs	12 hrs	15 hrs
tmax (h)	0.5-1 / 5		1-2 / 4	4-6	5-6	7	2
T1/2 (h)	2-4 / 16		3 / 6-8	45-55	20	3	15
Bioavailability in urine	<0.1%	<0.1%	15%	15%	60%	3%	<1%
Receptor subtype	Relatively M3 selective	Relatively M3 selective	Non-selective M3/M2	Relatively M3 selective	Non-selective M3/M2	M3 selective	Non-selective M3/M2
Dose available	IR 5mg, ER 5, 10, 15 mg	3.9 mg patch	IR 1, 2mg, ER 4mg	5, 10, 15, 20, 30 mg	20mg	7.5mg, 15mg	15mg, 30mg
Adjustment for renal or hepatic impairment	No	No	Max 1mg bd for severe renal impairment Avoid use in hepatic impairment	Max 5 mg /day for renal and hepatic impairment	Max 20 mg/ day for severe renal impairment avoid in hepatic impairment	7.5 mg/d for moderate hepatic impairment	Caution for 30mg/day if eGFR <30mL/min/1.73 Avoid in moderate and severe hepatic failure

### Clinical efficacy of antimuscarinics

The number of antimuscarinics available is increasing and placebo-controlled, double-blind, randomized clinical trials have shown efficacy in OAB.[43–49] Head to head trials pitching one against the other show effectiveness in providing relief to different symptoms within the OAB spectrum.[32,45,46,50–57] Systematic meta-analysis, published in recent years, have reviewed placebo-controlled, as well as the active-controlled trails; and commented on the efficacy of antimuscarinics based on urgency, incontinence episodes, frequency of micturition, voided volume per micturition, impact on quality of life along with tolerability, and side effects.[58,59]

Herbison *et al*. identified 64 potential trials, the 32 included were randomized placebo controlled trials published between January 1966 and January 2002.[58] Primary outcomes were patient’s observations of cure or improvement in symptoms, number of leakages, and number of voids. Secondary outcomes were urodynamic measures and adverse events. Quality of life and economic outcomes were also considered. Weighted mean differences in: change in incontinence episodes per 24 hours, micturitions per 24 hours, and maximum cystometric capacity were calculated. Patients receiving antimuscarinics reported a weighted mean difference (vs. placebo) of 0.56 (95% CI − 0.93 to − 0.15) for a change in incontinence episodes and 0.59 (95% CI − 0.83 to − 0.36) for the change in micturitions per 24 hours.

The changes in maximum cystometric capacity at a weighted mean difference (95% CI) of + 54 mls (43-66), also favored active treatment. The volume at first bladder contraction had a weighted mean difference of 52.25 (95% CI 37.45 to 67.06). Although these were statistically significant differences and favored antimuscarinic treatment over placebo, the authors challenged the clinical significance of the efficacy quoted because the magnitude of improvement in these endpoints was small.

Chapple and colleagues did a systematic review to assess the efficacy, safety and tolerability of antimuscarinic treatments for OAB and also to consider the effects of antimuscarinics on quality of life (QoL); and to assess whether there are differences between individual antimuscarinics.[59] Data from 56 blinded, randomized, placebo and active-controlled trials of oral and transdermal antimuscarinics used to treat OAB was collected and compared, with, comparisons between individual antimuscarinics The weighted mean difference (95% CI) vs. placebo for the change in incontinence episodes per 24 hours was significant for oxybutynin immediate release (IR) (15.0 mg/day; − 0.72, − 1.09 to − 0.34), transdermal oxybutynin (− 0.55, − 1.05 to − 0.04), solifenacin (5 mg/day; − 0.66, − 1.13 to − 0.19; 10 mg/day, − 0.69, − 1.19 to − 0.19), tolterodine IR (4 mg/day, − 0.50, − 0.70 to − 0.30), and tolterodine extended release (ER 4mg/day) (− 0.73, − 0.93 to − 0.53). For changes in micturition frequency per 24 h, there were significant improvements vs. placebo for transdermal oxybutynin (− 0.55, − 1.03 to − 0.07), solifenacin (5 mg/day, − 0.99, − 1.52 to − 0.46; 10 mg/day, − 1.41, − 1.97 to − 0.85), tolterodine IR (2 mg/day, −0.68, − 1.15 to − 0.22; 4 mg/ day, − 0.67, − 0.92 to − 0.42), and tolterodine ER 4 mg/ day (− 0.73, − 0.96 to − 0.49).

Some evidence suggesting propiverine IR and ER might also reduce incontinence episodes and micturition frequency relative to placebo; was not suitable for inclusion in the meta-analysis. The changes in urgency episodes per 24 hours vs. placebo were significant for solifenacin (5 and 10 mg/day) and tolterodine ER. Improvements in volume voided per micturition were significant for oxybutynin IR (8.8–15.0 mg/day), transdermal oxybutynin, solifenacin (5 mg and 10 mg/day), tolterodine IR (2 mg and 4 mg/day), and tolterodine ER. The ‘relative risk’, (RR, 95% CI) for return to continence was significant for oxybutynin IR (5.0–7.5 mg/day; 3.53, 1.94–6.41), transdermal oxybutynin (1.75, 1.16–2.62), tolterodine ER (1.72, 1.14–2.58), and trospium (2.00, 1.40–2.86).

This suggests that patients who receive antimuscarinics are nearly twice as likely to return to continence compared with placebo. QoL data were available for transdermal oxybutynin, tolterodine IR (4 mg/day), tolterodine ER, and trospium. The meta-analysis revealed significant weighted mean differences vs placebo for 27 of 37 QoL domains.[58] A meta-analysis of three active-controlled studies of tolterodine ER and tolterodine IR revealed no significant differences in the change in QoL measures between active treatments.[60]

Evidence from the 24 active-controlled trials included in the meta-analysis, suggests that patients receiving solifenacin (5 or 10 mg/day) had up to one fewer urgency episodes per day than did those receiving tolterodine IR (4 mg/day). Solifenacin (10 mg/ day) was also associated with significantly fewer micturitions per 24 hours compared to tolterodine IR (4 mg/day). Oxybutynin ER (10 mg/day) was associated with approximately two fewer incontinence episodes per week with a greater percentage of patients returning to continence when compared with tolterodine ER (4 mg/day) Patients treated with oxybutynin IR (15 mg/ day) or solifenacin (5 and 10 mg/day) compared with patients receiving tolterodine IR (4 mg/day) had a significant increase in voided volumes.[59]

Fesoterodine is a newer non-selective agent that acts as a competitive muscarinic receptor antagonist. It is functionally a prodrug and hydrolyzed to its active metabolite 5 – hydroxymethyl tolterodine and available in 4 mg and 8 mg formulations. Its active metabolite is similar to that of tolterodine. In phase II and III trials, fesoterodine has shown efficacy in improving the symptoms of OAB in patients who were previously dissatisfied with tolterodine or tolterodine ER.[61–63]

### Adverse effects and withdrawals

Dry mouth, constipation, headache, and blurred vision are frequent adverse effects in patients treated with oxybutynin, tolterodine, trospium, propiverine, darifenacin, and solifenacin in clinical trials.[43–49,64–71] In placebo- and active-controlled trials, the incidence of dry mouth in patients taking oxybutynin IR was 17–97%, oxybutynin ER 23–68%, transdermal oxybutynin 4–39%, propiverine IR 20–47%, propiverine ER 22%, tolterodine IR 8–50%, tolterodine ER 7–34%, trospium 3–41%, solifenacin 8–30%, and darifenacin 18–31%; a dry mouth is common and exceeded the combined incidence of constipation, headache, and abnormal vision.[43–57] Oxybutynin IR was found to be associated with a greater incidence of dry mouth compared with oxybutynin ER, oxybutynin transdermal, propiverine IR, tolterodine ER, tolterodine IR and trospium.[59] The Relative risk (95% CI) for dry mouth was significantly greater than placebo for patients receiving antimuscarinics with the exceptions of low dose oxybutynin IR (5.0–7.5 mg/day; 1.06, 0.90–1.29), transdermal oxybutynin (1.35, 0.67–2.72), and propiverine IR (45 mg/day; 3.00, 0.13–71.9).[49] Transdermal oxybutynin circumvents ‘first-pass’ metabolism and results in lower concentrations of the active metabolite N-desethyloxybutynin. This metabolite has a greater affinity for muscarinic receptors in the parotid gland and might contribute to dry mouth reported by many patients receiving oral oxybutynin.[42,43]

Other reported adverse effects are blurred vision (with oxybutynin IR, propiverine IR and solifenacin); constipation (with darifenacin, solifenacin and trospium); dyspepsia (with darifenacin and oxybutynin IR); erythema and pruritus (with oxybutynin TDS); and urinary retention (with oxybutynin IR). Solifenacin 10 mg/day was associated with a higher incidence of blurred vision than tolterodine IR 4 mg/day; the risk of constipation was reduced by using oxybutynin transdermal in preference to oxybutynin IR, and tolterodine IR in preference to solifenacin nausea was less in patients treated with oxybutynin ER compared with oxybutynin IR; and the risk of vomiting was less for oxybutynin ER compared with tolterodine ER. With low incidence it is difficult to validate these differences.

There have been concerns raised with an increased risk of cardiac adverse events with antimuscarinics, particularly increases in heart rate and QT prolongation and induction of polymorphic ventricular tachycardia (torsade de pointes); however, at recommended therapeutic doses there is no evidence to substantiate this concern.[10,46,72,73]

Withdrawal due to adverse events has been infrequent in placebo- and active-controlled trials of oral antimuscarinic. Herbison *et al*. reported that patients receiving placebo were equally likely to withdraw owing to adverse events as were those receiving antimuscarinics (RR 1.01, 95% CI, 0.78–1.31).[58] In the Chapple meta-analysis, the RR (95% CI) for all-cause withdrawals was significantly greater in patients receiving oxybutynin IR (8.8–15.0 mg/day, 1.40, 1.06–1.84) compared with placebo and the risk of all-cause withdrawal for patients receiving tolterodine ER (0.71, 0.51–0.99) was significantly less than placebo.[59]

The difference with other antimuscarinic agents and placebo did not reach statistical significance Tolterodine IR was found to cause fewer withdrawals due to adverse events than oxybutynin IR and; tolterodine ER caused fewer than both oxybutynin IR and transdermal oxybutynin. Literature supports the tolerability of antimuscarinics for the treatment of OAB symptoms with adverse event profiles dependent on muscarinic receptor subtype selectivity. In the clinical scenario, should the patient wish to discontinue treatment with one type of antimuscarinic, it is worth trying a different drug as this might be as effective and better tolerated.

### Antimuscarinics in special conditions OAB with bladder outlet obstruction

There has been some concern that the inhibitory effect of antimuscarinics on detrusor muscle contraction could theoretically impair detrusor contractility and thus cause urinary retention in men with OAB symptoms secondary to BOO.

Athanasopoulos *et al*. reported on a randomized study of 50 men aged 52-80 years with mild to moderate bladder outlet obstruction and proven detrusor overactivity; comparing tamsulosin with a combination therapy of tamsulosin and tolterodine over a three-month period. There were no episodes of retention in the either group and the addition of tolterodine to tamsulosin is safe; patients with severe obstruction were excluded from this trial.[74]

Abrams *et al*. in a double-blind, placebo-controlled study, reported on the efficacy of antimuscarinics monotherapy in patients with symptoms of OAB and urodynamic evidence of DO and BOO.[75] A total of 222 men were recruited over 12-week and randomized 2:1 between treatments with tolterodine 2 mg twice daily or a placebo. Urodynamic parameters including change in Q_max_ and detrusor pressure at Q_max_ were comparable in the two groups, while volume to first detrusor contraction (reflex volume) and maximum cystometric capacity were significantly improved in men receiving tolterodine. A statistically significant increase in PVR (+33 mL vs. placebo) was not considered clinically significant; and micturition disorders led to withdrawals in one of 72 (1%) patients receiving placebo and three of 150 (2%) patients receiving tolterodine.

In a recent blinded randomized prospective trial, Kaplan *et al*. studied the efficacy of tolterodine ER and/or the alpha1-receptor antagonist tamsulosin in 879 men who met research criteria for both OAB and benign prostate hypertrophy.[76] A significantly greater proportion of patients receiving combined therapy (80%) reported treatment benefit by week 12 compared with placebo (62%), tamsulosin (71%), or tolterodine ER (65%). Combined therapy resulted in significant improvements in bladder diary variables (i.e. UUI, urgency, 24-hour micturition frequency, and nocturnal frequency) and the IPSS total and QoL scores vs. placebo. The incidence of AUR requiring catheterization was low (tolterodine ER plus tamsulosin, 0.4%; tolterodine ER, 0.5%; tamsulosin, 0%; placebo, 0%).

Recent evidence suggests that the incidence of acute retention in patients with bladder outlet obstruction and on antimuscarinics is low. However, their safety in patients with larger residues and over long term treatment needs to be established.

### Antimuscarinics in elderly patients

Impairment of the cognitive function in the elderly with the use of antimuscarinic is of particular concern. All five muscarinic receptor subtypes have been identified in brain tissue and a decline in CNS cholinergic activity has been observed with normal ageing possibly due to a reduction in the density of functioning muscarinic receptors. A large-scale study by Staskin *et al*., in patients with OAB showed no significant differences in measures of sleepiness or CNS adverse effects between 20 mg trospium and placebo.[77] Trospium did not increase the daytime sleepiness or affect the alertness levels across the age groups.

Kay *et al*. studied the effects of darifenacin and oxybutynin on cognitive function of older patients.[78] 150 patients 60 years or older were randomized to darifenacin, oxybutynin ER or placebo. At three weeks, darifenacin did not show any significant difference as compared to a placebo (mean difference = -0.06, *P* = 0.908) on the Name-Face Association test. Whereas oxybutynin ER resulted in memory impairment, with significant lower scores than placebo and darifenacin (mean differences = -1.30, *P* = 0.011 and -1.24, *P* = 0.022 respectively).

Although reports of hallucinations in elderly high-risk patients using tolterodine have been published, a causal relationship between tolterodine treatment and cognitive impairment needs to be established.[79,80] Higher-quality studies, such as randomized controlled trials, prospective epidemiological studies, and large case series, are required to determine if any causal relationship exists between antimuscarinic treatment and cognitive impairment.

### Antimuscarinics in neuropathic bladder

Propiverine hydrochloride is one of the few drugs recommended for the treatment of detrusor overactivity by the International Consultation on Incontinence.[81] It comprises a neurotropic and a musculotropic mode of action, thus inducing antimuscarinic effects as well as effects on the calcium influx and calcium-homeostasis.[82] In a dosage-optimizing study of spinal cord injured adults, Mazur and coworkers recommended 15 mg propiverine thrice daily dose as being adequate in most patients.[83] Subsequently, Stohrer *et al*. proved the efficacy of propiverine (15 mg thrice daily) compared to placebo over a treatment period of 14 days by documenting urodynamic improvements.[84]

In a multicenter placebo-controlled double-blind study 61 patients with spinal cord injuries and detrusor hyperreflexia were treated with 20 mg trospium chloride twice daily over a period of 3 weeks. Pre- and post treatment urodynamic measurements demonstrated large improvements in maximum cystometric capacity (mean = 138.1 ml), decreased maximum detrusor pressure (mean = -37.8 cm H_2_O) and an increase in compliance (mean = 12.1 ml/cm H_2_O) in the treatment group. Significant difference for these parameters was reported with, no effect on maximum flow rate and residual urine.[85]

Currently only propiverine and trospium are licensed to be used for OAB in a neuropathic bladder but trials have been done to assess the efficacy of other anticholinergics in these patients. In patients with neurogenic detrusor overactivity, mainly due to traumatic spinal cord injury, a randomized controlled trial, comparing propiverine and oxybutynin, showed they were equally effective, both for the medical objective of lowering detrusor pressure and for the patient objective of achieving continence.[86] However, patients on propiverine showed fewer anticholinergic adverse events, especially dryness of the mouth.

## FUTURE TRENDS

The market with antimuscarinic pharmacotherapy is lucrative with many players in the industry constantly investigating newer molecules with greater efficacy and fewer side effects; the race to find the next blockbuster is on. Improved uroselectivity might be achieved through alterations in structure or route of administration.[34] New drug classes under investigation for OAB include α3-adrenoceptor agonists, purinergic receptor antagonists, phosphodiesterase inhibitors, neurokinin-1 receptor antagonists, opioids, and Rho-kinase inhibitors.[34] However, clinical data, particularly comparing efficacy and safety is awaited before clinical use.

## CONCLUSIONS

OAB syndrome is a very common condition that affects people of all ages and may cause a severe impact on quality of life. OAB can be diagnosed easily and managed effectively with both non-pharmacological and pharmacological therapies. The first-line treatments are lifestyle interventions, bladder training, pelvic floor muscle exercises and anticholinergic drugs. Antimuscarinics are the drug class of choice for OAB symptoms; with proven efficacy, and adverse event profiles that differ somewhat. Physicians can individualize OAB treatment by working with each patient to identify the most effective and best tolerated agent. Failure with one agent does not preclude usage of another and recently combinations of anticholinergics with improved efficacy and no increase in side-effects have been reported.
